# Hepatitis of unknown etiology in children: What we know and what we can do?

**DOI:** 10.3389/fmicb.2022.956887

**Published:** 2022-08-08

**Authors:** Mingyu Zhu, Li Chen

**Affiliations:** Department of Gastroenterology, Ruijin Hospital, Shanghai Jiao Tong University, School of Medicine, Shanghai, China

**Keywords:** acute hepatitis of unknown etiology, SARS-CoV-2, adenovirus, working hypotheses, multisystem inflammatory syndrome

## Abstract

Recently, acute hepatitis of unknown etiology in children has gained great concern since March 2022. The disease was first reported by Public Health Scotland. Cases increased rapidly and are now reported in 33 countries worldwide. All cases are predominantly aged under 5 years old. Most patients presented with jaundice, and remarkably, some cases progress to acute liver failure. Until now, the etiology is not fully elucidated, and the investigations are ongoing. Adenovirus infection seems to be an important factor. Several hypotheses on the etiology have been proposed. This review aims to summarize current research progress and put forward some suggestions.

## Introduction

On 31 March 2022, Public Health Scotland was first alerted to five children aged 3–5 years admitted to the Royal hospital with severe acute hepatitis of unknown etiology within 3 weeks (Marsh et al., 2022). In the following weeks, more cases were reported in the United Kingdom, the United States, and European countries (Baker et al., 2022; European Centre for Disease Prevention and Control, 2022a; UK Health Security Agency, 2022a). Most cases are aged under 5 years old. Jaundice and gastrointestinal symptoms are the main clinical manifestations. All cases presented with markedly elevated transaminases, and remarkably, some cases progress to acute liver failure and even die. So far, common hepatotropic viruses have been ruled out and no definite cause has been identified. In epidemiological terms, cases are almost sporadic but widely distributed (World Health Organization, 2022a). Given the severity of the disease and the unascertained pathogenesis, this is an urgent issue that should be paid more attention to worldwide. Here, we would like to discuss what we know about acute hepatitis of unknown etiology so far and what we can do next.

## What do we know?

### Epidemiology

**FIGURE 1:**
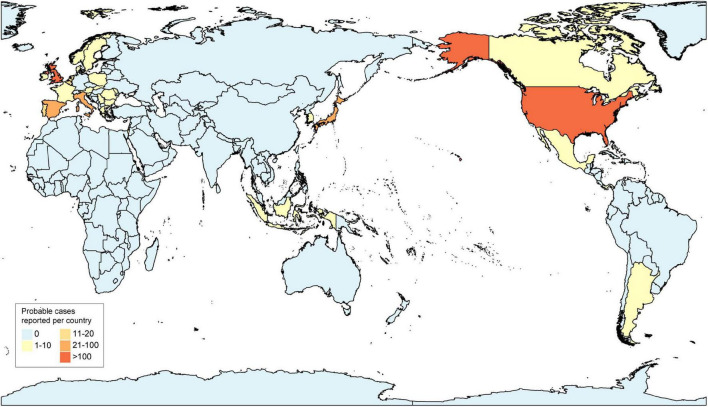
Distribution of probable cases of acute hepatitis of unknown etiology by country, as of 26 May 2022.

Since mid-April 2022, the UK Health Security Agency (UKHSA), the European Centre for Disease Prevention and Control (ECDC), and the World Health Organization (WHO) are closely monitoring cases of hepatitis of unknown etiology and to define the criteria for diagnosis in different countries and territories to report cases and to investigate potential causes. As of 26 May 2022, the total number of cases reported worldwide is approximately 650, including at least 14 deaths (European Centre for Disease Prevention and Control, 2022b; World Health Organization, 2022b). A total of 222 cases have been identified in the United Kingdom, and approximately 430 cases have been identified in 18 EU/EEA countries (Austria (<5), Belgium (14), Bulgaria (<5), Cyprus (<5), Denmark (7), France (<5), Greece (<5), Ireland (7), Italy (27), Netherlands (14), Norway (<5), Poland (<5), Portugal (11), Romania (<5), Slovakia (<5), Slovenia (<5), Spain (29), Sweden (9), and 14 other countries (Argentina (<5), Canada (10), Indonesia (<5), Israel (12), Japan (31), Maldives (<5), Mexico (10), the Republic of Moldova (<5), Occupied Palestinian Territories (<5), Panama (<5), the Republic of Korea (<5), Serbia (<5), Singapore (<5), and the United States of America (216) (World Health Organization, 2022b; Figure 1). From the report of Scotland, two children were in close contact with two other cases and, so far, no epidemiological links have been reported except for two pairs of cases (Marsh et al., 2022), and no related cases have been reported in China.

### Clinical characteristics

The definition of acute hepatitis in children of unknown etiology has changed as investigations have progressed. All the definitions in different regions and periods were summarized in Table 1 (UK Health Security Agency, 2022a). Most of the initial cases reported in Scotland were presented with serum levels of transaminase greater than 2,000 international units per liter (IU/L) and hepatitis virus infection (A to E) has been excluded (Marsh et al., 2022). So far, from the investigation of trawling questionnaires, no common environmental exposures in travel, family structure, parental occupation, diet, water source, or potential exposures to toxicants have been found (UK Health Security Agency, 2022b). The etiology needs further investigation. Coincidentally, clinicians had noticed similar cases from October to November 2021 in Alabama, and the report on these cases was published in April 2022 (Baker et al., 2022). Due to uneven medical conditions in different countries around the world, we can speculate that such cases may have occurred earlier than October 2021, but these have not been taken seriously or recognized.

**TABLE 1:** The definition of acute hepatitis in children of unknown etiology.

Case definition	Description
**Scotland (in the technical briefing 1 on 23 April 2022)**	
Confirmed	A person presenting with a serum transaminase greater than 500 IU/L (AST or ALT) without any known cause, who is 10 years of age and under or a contact of any age of a possible or confirmed case, since 1 January 2022.
Possible	A person presenting with jaundice without any known cause, who is 10 years and under or contact of any age to a possible or confirmed case, since 1 January 2022.
**England, Wales, Northern Ireland (in the technical briefing 1 on 23 April 2022)**	
Confirmed	A person presenting with an acute hepatitis (non-hep A-E*) with serum transaminase > 500 IU/L (Aspartate Transaminase-AST or Alanine Transaminase -ALT), who is 10 years old and under, since 1 January 2022.
Possible	A person presenting with an acute hepatitis (non-hep A-E*) with serum transaminase > 500 IU/L (AST or ALT), who is 11–16 years old, since 1 January 2022.
Epi-linked	A person presenting with an acute hepatitis (non-hep A-E*) of any age who is a close contact of a confirmed case, since 1 January 2022.
**Scotland (in the technical briefing 2 on 6 May 2022)**	
Confirmed	A person presenting with a serum transaminase greater than 500 IU/L (AST or ALT) without any known cause (excluding hepatitis A-E, Cytomegalovirus and Epstein-Barr Virus), who is 10 years of age and under or a contact of any age of a confirmed case, since 1 January 2022.
**England, Wales, Northern Ireland (in the technical briefing 2 on 6 May 2022)**	
Confirmed	A person presenting since 1 January 2022 with an acute hepatitis which is not due to hepatitis A-E viruses, or an expected presentation of metabolic, inherited or genetic, congenital or mechanical cause** with serum transaminase greater than 500 IU/L (AST or ALT), who is 10 years old and under.
Possible	A person presenting with an acute hepatitis since 1 January 2022 with an acute hepatitis which is not due to hepatitis A-E viruses or an expected presentation of metabolic, inherited or genetic, congenital or mechanical cause** with serum transaminase greater than 500 IU/L (AST or ALT), who is 11–15 years old.
Epi-linked	A person presenting since 1 January 2022 with an acute hepatitis (non-hepatitis A-E) who is a close contact of a confirmed case. (A person who is epi-linked but also meets the confirmed or possible case definition will be recorded as a confirmed or possible case and their epi-link noted in their record. This prevents double-counting of cases).
**WHO and ECDC**	
Confirmed	N/A
Probable	A person presenting with an acute hepatitis (non-hepatitis viruses A, B, C, D, and E*) with aspartate transaminase (AST) or alanine transaminase (ALT) over 500 IU/L, who is 16 years old or younger, since 1 October 2021.
Epi-linked	A person presenting with an acute hepatitis (non-hepatitis viruses A, B, C, D, and E*) of any age who is a close contact of a probable case since 1 October 2021.

*If hepatitis A-E serology results are awaited, but other criteria were met, these are classified as “pending classification.”

**Confirmed and possible cases should be reported based on clinical judgment if some hepatitis A-E virus results are awaited, or if there is an acute on chronic hepatic presentation with a metabolic, inherited or genetic, congenital, mechanical, or other underlying cause. If hepatitis A-E serology results are awaited, but other criteria were met, these will be classified as “pending classification.”

In the description of the technical briefing 3 published by the UKHSA, 50% of England cases are female and the majority are of white ethnicity (86.3%). They are predominantly aged between 3 and 5 years old (53.5%), with a median age of 3 years (interquartile range 2–4 years). The main symptoms in these children are shown in Table 2. Notably, they were reported to be immunocompetent before this admission (UK Health Security Agency, 2022b). As of 26 May 2022, 14 cases have undergone liver transplantation (World Health Organization, 2022b).

**TABLE 2:** The main symptoms in UK cases with acute hepatitis of unknown etiology.

Symptoms	Number of patients (%)
Jaundice Vomiting Lethargy Diarrhea Pale stools Abdominal pain Fever Nausea Respiratory symptoms	68.8 57.6 48.6 43.1 42.7 36.1 28.5 25.7 18.1

### Histopathologic characteristics

The hepatotropic viruses (hepatitis A, B, C, D, and E viruses) have been excluded and, therefore, the non-hepatotropic viruses, such as cytomegalovirus (CMV), Epstein-Barr virus (EBV), herpes simplex virus (HSV), human herpesvirus-6 (HHV-6), adenovirus, echovirus, etc., should be considered. Generally, the histological features of acute hepatitis in children mainly manifested as lobular hepatocellular injury, inflammatory infiltrate with lobular predominance, and portal or periportal infiltrate, and viral inclusions are specific features of non-hepatotropic viruses related to hepatitis (White and Dehner, 2004). Certain histologic characteristics of liver injury may be suspicious for a specific agent. For instance, for CMV hepatitis, a paucity of bile ducts may be present. Nonzonal confluent necrosis and nuclear target-like inclusions in hepatocytes are biopsy features of adenovirus-related hepatitis (White and Dehner, 2004).

To further clarify the etiology, some patients received a hepatic histopathologic assessment. In the UK, liver specimens included 8 biopsies and 6 liver tissue samples obtained from the children receiving liver transplantation. Variable severity ranging from mild hepatocellular injury to massive hepatic necrosis was found in the histopathologic evaluation. The histopathologic pattern showed non-specific changes (UK Health Security Agency, 2022a). In Alabama, United States, liver biopsies from six children presented various degrees of hepatitis. Moreover, immunohistochemical (IHC) evidence of adenovirus cannot be observed and viral particles cannot be identified by electron microscopy (Baker et al., 2022). However, viral inclusions may not be obvious in all cases. At present, laboratory tests and histological examination cannot ascertain the etiology.

### Etiological findings

Considering the epidemiological and clinical features, an infectious etiology may be the most possible cause, then all patients were tested for pathogens at or around the time of admission. The possible infection-causing factors were presented in Table 3. The adenovirus is the most frequent pathogen detected in various samples. Among 197 UK cases, 68% (116 of 179 cases with available results) of cases have tested positive for adenovirus (UK Health Security Agency, 2022b). Adenovirus viral loads in blood or serum samples in those who received liver transplantation were approximately 12-fold higher than in those who did not (UK Health Security Agency, 2022b). In the study, Adenovirus was detected more frequently in blood or serum samples (79.4%) than in stool (43.9%) or respiratory (27.3%) samples (UK Health Security Agency, 2022b). So, cases that were not tested on whole blood or serum samples showed negative results, which may lead to an underestimation of positivity for adenovirus. By partial Hexon gene sequencing, 35 cases have been successfully subtyped, of which 27 (77%) cases are adenovirus type 41 (UK Health Security Agency, 2022b). Among 9 American cases, adenovirus type 41 was detected in all patients (Baker et al., 2022).

**TABLE 3:** Summary of the possible infectious causing factors observed in cases of hepatitis of unknown etiology in different countries.

Country	Adenovirus (%, cases)	SARS-CoV-2 (%, cases)	Other positive results (cases or samples)
The United Kingdom	68% (116/179) 77% (27/35) Type 41	15% (25/169)	HHV-6 20/(60–70) HHV-7 (10–20)/(50–60) EBV (10–20)/(130–140) Enterovirus (10–20)/(70–80) CMV (5–10)/(130–140) RSV (<5)/(70–80) adeno-associated dependoparvovirus A (13 samples), Human Polyomavirus (4 samples) AAV2 (9 samples)**
The United States of America*	100% (9/9), all Type 41	0/9	EBV 6/9 Enterovirus/rhinovirus 4/8 Metapneumovirus 1/8 Respiratory syncytial virus 1/8 Human coronavirus OC43 1/8
Israel	NA	91.7% (11/12)	NA

SARS-CoV-2, severe acute respiratory syndrome coronavirus 2; HHV, human herpesvirus; EBV, Epstein-Barr virus; CMV,: Cytomegalovirus; RSV, Respiratory Syncytial Virus NA, no available data at the time of publication; AAV2, adeno-associated virus 2.

*These results are available in the study of Alabama, but there is no information on the reported cases later in other regions of America.

**HHV-6, HHV-7, EBV, Enterovirus, CMV, and RSV are represented by the number range of cases because we cannot obtain the exact number in the reports of UKHSA. Associated dependoparvovirus A, Human Polyomavirus, and AAV2 are represented by the samples because these viruses are detected by metagenomic sequencing.

The SARS-CoV-2 was also frequently detected in reported cases. In the United Kingdom, SARS-CoV-2 has been detected in 25 cases of 169 with available results (15%) (UK Health Security Agency, 2022b). From the report of Israel, 11 of 12 patients had SARS-CoV-2 infection in recent months (Haaretz, 2022). From the WGS of SARS-CoV-2 in England cases, 5 sequences are classified as VOC-22JAN-01 (lineage BA.2) (UK Health Security Agency, 2022a). Seven cases were co-infected with adenovirus and SARS-CoV-2 (PCR or lateral flow device) and serological testing is underway (UK Health Security Agency, 2022a).

Other pathogens, such as enterovirus, parechovirus, human herpesvirus 6 and 7 (HHV-6, HHV-7), and varicella-zoster virus, have been detected in some UK cases (UK Health Security Agency, 2022a). Of 9 Alabama cases, 6 cases showed positive test results for EBV by PCR but negative results for EBV immunoglobulin M (IgM) antibodies, suggesting low-level reactivation of previous infections rather than acute infection. Enterovirus/Rhinovirus, metapneumovirus, respiratory syncytial virus, and human coronavirus OC43 have also been detected (Baker et al., 2022). In addition, preliminary metagenomics findings that Adeno-associated virus 2 (AAV2), Adeno-associated dependoparvovirus A, Human Herpes Virus, and Human Polyomavirus detected in samples from cases in England and Scotland were published on 19 May 2022 (UK Health Security Agency, 2022b).

### Possible mechanisms

Based on the working hypotheses put forward by the UKHSA and the views of experts, we summarize the possible mechanisms as follows.

#### Human adenovirus infection

Adenoviruses are a group of double-stranded non-enveloped DNA viruses. They consist of 7 species (A–G) and are currently classified into 51 serologically distinct types (Lynch and Kajon, 2016). Different types of adenoviruses show various tissue tropisms, and a certain type is often associated with a particular clinical manifestation. Acute respiratory symptoms are the most common manifestations (serotypes 1–5, 7, 14, and 21). Besides, keratoconjunctivitis (serotypes 8, 19, and 37), urethritis (serotypes 11, 34, 35, 3, 7, and 21), and gastroenteritis (serotype 40 and 41) are also important symptoms (Ronan et al., 2014; Lynch and Kajon, 2016). Type 41 adenovirus infection often presents with gastrointestinal disease, for example, vomiting and diarrhea, and it is a common cause of pediatric acute gastroenteritis (Lynch and Kajon, 2016). Acute hepatitis is an uncommon manifestation of adenovirus infection. However, a few cases were reported in both immunocompetent and immunosuppressed individuals (Lion, 2014). In addition, adenovirus infection was identified as the causing factor of acute liver failure in several case reports (Ozbay Hosnut et al., 2008; Gu et al., 2021).

In previous studies, pediatric acute liver failure (PALF) is a rare but rapidly progressive and life-threatening disorder, and approximately 22–49% of cases were diagnosed with indeterminate etiology (Squires et al., 2006; Berardi et al., 2020). The PALF study is a multicenter, observational cohort study and it demonstrated that 46% of cases with acute liver failure of unknown etiology undergo liver transplantation, which is higher than the group with definite etiology (Squires et al., 2006). As of 26 May 2022, 14 (2%) cases have undergone liver transplantation and at least 24 (4%) cases are waiting for liver transplantation in recently reported acute hepatitis of unknown etiology (World Health Organization, 2022b). Acute hepatitis of unknown etiology in children is not a newly emerging disease. However, the higher-than-expected numbers in 2022 require more attention, especially during the current COVID-19 pandemic worldwide. COVID-19 prevention and control measures, such as physical distancing restrictions, could result in the lack of opportunity for exposure to pathogens for younger children. These children may be relatively immunologically naïve and more susceptible to adenovirus infection and, unexpectedly, severe effects may occur (Samarasekera, 2022). With the decline of pandemic restrictions, more social mixing leads to children’s first exposure to pathogens, allowing a rarer outcome of adenovirus infection to be detected. Retrospective analysis of the trends of adenovirus infection in England from 1 January 2017 to 1 May 2022 showed a reduction in the number of adenovirus cases between March 2020 and May 2021 (COVID-19 pandemic period) and a remarkable exceedance of adenovirus infection in children under 10 years old since the end of 2021 (UK Health Security Agency, 2022a). After a period of a low prevalence of adenovirus infections during the previous 2 years of the COVID-19 pandemic, a massive wave of adenovirus infections may emerge in a short time and a serious outbreak may happen. According to the clinical manifestations and outcomes of the present cases, although most of the cases recovered after supportive treatment, the level of transaminases in reported cases was higher than that in general hepatitis. Moreover, approximately 6 percent of cases progress to acute liver failure (Marsh et al., 2022). In addition, re-infection with adenovirus after SARS-CoV-2 infection, or coinfection of SARS-CoV-2 and adenovirus, may make the clinical outcome worse (The Lancet Infectious Diseases, 2022). However, only seven cases (5.6%, 7/125) were co-infected with adenovirus and SARS-CoV-2 in England, and the proportion is not high. This hypothesis needs to be further verified.

Another hypothesis is adenovirus mutation. The molecular evolution of Adenovirus by homologous recombination can result in novel viruses showing various tissue tropisms and increased virulence (Lion, 2014; Tian et al., 2019). Whole-genome sequencing (WGS) of adenovirus facilitates the discovery of viral recombinants. The virulence and clinical manifestations of the novel recombinant virus may differ significantly from those of adenoviruses previously reported. Thus, firm conclusions can be drawn after WGS. Noteworthy, the low level of adenovirus present in blood samples suggests that the data quality may affect the results of WGS. The investigation is ongoing.

However, the evidence, of which adenovirus seems to be a decisive causative factor, was still limited. Firstly, the viral load of adenovirus present in blood samples is not high (UK Health Security Agency, 2022a). Secondly, viral inclusions, IHC evidence of adenovirus, or viral particles were not identified in the liver biopsies of related cases until now. One case successfully underwent adenovirus PCR of liver tissue, but the result was negative (Baker et al., 2022; UK Health Security Agency, 2022a). It seems uncertain whether adenoviruses are incidental concomitant or play important role in acute hepatitis.

#### SARS-CoV-2 infection or multisystem inflammatory syndrome

Firstly, acute hepatitis in children may be the post-infectious sequelae of SARS-CoV-2 infection. Studies in adults showed that elevated serum alanine aminotransferase (ALT) and aspartate aminotransferase (AST) levels during SARS-CoV-2 infection are common manifestations (Marjot et al., 2021). The level of ALT and AST varies in different patients. The underlying causes of elevated liver enzymes in SARS-CoV-2 infection is temporarily not clear. Direct infection of hepatocytes and immune-mediated inflammatory response may be both vital in the progress of the liver injury. After SARS-CoV-2 infection, the SARS-CoV-2 spike protein will bind to the human angiotensin-converting enzyme 2 (ACE2) receptor for entry into the target cell, then trigger an immune response in the host. The innate immune system will work together with T and B cells to control SARS-CoV-2. Patients exhibit enhanced pro-inflammatory responses and corresponding organ dysfunction and symptoms. Differences in antibodies, CD4^+^ and CD8^+^ T cell populations, genetic polymorphism in MHC, and cytokine production will result in different clinical outcomes (Shah et al., 2020; Fergie and Srivastava, 2021). In a recent study preprinted in medRxiv, Kendall et al. (2022) reported that children infected with SARS-CoV-2 are at significantly increased risk for elevated AST or ALT (hazard ratio or HR:2.52, 95% confidence interval or CI: 2.03–3.12) and total bilirubin (HR: 3.35, 95% CI: 2.16–5.18), compared to children infected with other respiratory infections. Liver manifestations and pathophysiological aspects related to SARS-CoV-2 infection in patients without liver diseases have been summarized in a recently published review. Even though transaminase elevation is common during COVID-19, severe acute liver injury is relatively rare. A “cytokine storm,” which is characterized by the release of a large number of inflammatory factors, plays a vital part in the pathophysiological of SARS-CoV-2 infection (Dufour et al., 2022). With a deeper investigation and understanding of COVID-19, several studies found that the activation and proliferation of a memory T-cell pool against SARS-CoV-2 contribute to rapid viral clearance during viral reinfection (Shah et al., 2020; Jung et al., 2021; Wang et al., 2021). SARS-CoV-2-specific CD4^+^ and CD8^+^ memory T-cell immunity can be detected not only in patients who recovered from COVID-19 but also in close contacts, who are SARS-CoV-2-exposed but are negative in nucleic acid and antibody screening (Wang et al., 2021). Moreover, SARS-CoV-2-specific memory T cell response would persist up to 10 months after infection (Jung et al., 2021). In the indeterminate PALF patients, IHC staining for liver tissue sections showed that the tissue-resident memory CD8^+^ T-cells are predominantly infiltrated leukocytes, which were responsible for the aberrant immune activation during liver failure (Chapin et al., 2018). Therefore, we can speculate that the children with acute hepatitis of unknown etiology may be in close contact with SARS-Cov-2 infection cases resulting in the generating of memory T cells response. At the reinfection of SARS-CoV-2, dysregulation of immune response can cause rapid liver damage, even acute liver failure (Bogunovic and Merad, 2021). Additionally, Osborn et al. (2022) reported a previously healthy 3-year-old female who develop acute liver failure secondary to type 2 autoimmune hepatitis (AIH) preceded by mild infection with SARS-CoV-2. This case showed a possible association between SARS-CoV-2 infection and subsequent development of autoimmune liver disease presenting with acute liver failure (Osborn et al., 2022). Although the mechanisms of AIH and acute hepatitis of unknown origin may be different, SARS-CoV-2 infection may play an important role in both cases.

Secondly, Staphylococcal enterotoxin B (SEB) is one of the most severe and typical bacterial superantigens that stimulate non-specific T cells and result in the release of large amounts of cytokines, leading to multi-organ system failure and death (Fries and Varshney, 2013). Yarovinsky et al. (2005) reported that the exposure of SEB to adenovirus-infected mice increases the severity of liver injury, in which the IFN-γ signaling and apoptosis may play important roles. Interestingly, structure-based computational models showed that the epitope of the SARS-CoV-2 spike protein has a sequence motif unique to SARS-CoV-2, which highly resembles the sequence and structure of SEB (Cheng et al., 2020). Therefore, Brodin (2022) recently proposed a hypothesis about SARS-CoV-2 superantigens that SARS-CoV-2 infection can cause viral reservoir formation. When SARS-CoV-2 continues to stimulate the gastrointestinal tract, a superantigen motif within the SARS-CoV-2 spike protein that resembles SEB will mediate the immune activation. This immune activation may result in multisystem inflammatory syndrome in children (MIS-C) (Cheng et al., 2020; Porritt et al., 2021; Brodin, 2022). Exposure to adenovirus after SARS-CoV-2 infection will lead to a broad and non-specific T-cell activation, resulting in apoptosis of hepatocytes. Immunomodulatory therapies would be suggested when superantigen-mediated immune activation is verified. Furthermore, several cases reported in Israel were treated with steroids and recovered (Haaretz, 2022), which indirectly suggested the role of immune-mediated inflammatory response.

Meanwhile, whether SARS-CoV-2 infection is vital in the progress of hepatitis remains controversial. The number of cases with SARS-CoV-2 coinfection is relatively less, while most cases have no history of SARS-CoV-2 infection. Serological investigations are ongoing to explore prior infection. Otherwise, in the report of Alabama, two of the cases that developed acute liver failure were treated with steroids, and medical treatment efficiency was limited (Baker et al., 2022). The hypothesis of SARS-CoV-2 mediated MIS-C is hard to explain in these cases.

There is no evidence of any correlation between hepatitis and the COVID-19 vaccine because the COVID-19 vaccine is not recommended for children under 5 years old, and nearly 75% of children with acute hepatitis of unknown etiology are under 5 years old and are too young to receive the vaccination (UK Health Security Agency, 2022a).

#### Host immune deficiency

In general, despite the increasing number of new cases, the prevalence of unknown hepatitis is relatively low. All cases presented with liver injury, but most of the cases recovered through supportive treatment, and only a small proportion of cases progressed to acute liver failure or death. Professor Kelly put forward a hypothesis that there may be genetic or immunologic differences, which make individuals more susceptible to a certain pathogen or activate a more potent immune response, between patients and healthy children (Samarasekera, 2022). Pathogen investigations are ongoing, and the immune state of the host is also of great importance. In the investigation planning of the UKHSA, the research on host characterization, including harmonized clinical data collation and analysis, host genetic characterization, immunological characterization including T cell activation studies, and transcriptomics will be launched. These studies will lead to a deeper understanding of the etiology.

## What we can do

### Diagnosis

The diagnosis of acute hepatitis of unknown etiology in children includes early recognition of symptoms and performing laboratory testing. In most cases, the onset of jaundice was preceded by gastrointestinal manifestations, such as diarrhea, nausea, and vomiting. Parents should strengthen the awareness of the children with corresponding symptoms and general practitioners, or other medical specialists should raise the awareness of this hepatitis of unknown etiology among young children. A person presenting with acute hepatitis with AST or ALT of over 500 IU/L, who is under 16 years old, needs further investigation. Clinical and epidemiologic characteristics of cases should be surveyed. The history of potential exposures to toxicants and drugs, food and water consumption, and the history of SARS-Cov-2 and other pathogen infections should be collected. Routine laboratory tests, including blood cell analysis, biochemical test, coagulation test, and plasma ammonia, the hepatotropic viruses (hepatitis A, B, C, D, and E viruses) and abdominal imaging examination should be performed. According to the definition of acute hepatitis in children of unknown etiology, a probable or epidemiologically linked (epi-linked) case can be identified.

### Monitoring and testing

A panel of tests is suggested to conduct when a probable or epi-linked case is identified. Adenovirus, enteroviruses, CMV, EBV, HSV, HHV6, HHV 7, and parechovirus should be tested in the blood. Respiratory virus (including influenza, adenovirus, parainfluenza, rhinovirus, respiratory syncytial virus, and human bocavirus 1–3), SARS-CoV-2, enteroviruses, and human metapneumovirus (hMPV) should be tested in throat swab specimens. Enteric viruses (including, norovirus, enteroviruses, rotavirus, astrovirus, and sapovirus) should be screened in the stool specimens. Also, according to the advice of ECDC, the serology of *Brucella* spp., Bartonella henselae, and Borrelia burgdorferi (if epidemiologically appropriate) should be tested. The culture of bacterial pathogens and viruses is an alternative valuable tool. Metagenomic analysis (blood and liver specimen) should be performed, and it is useful and meaningful. Other non-infectious causes (autoimmune hepatitis, Wilson disease, bacteremia, etc.) should also be considerably excluded. If necessary, a liver biopsy is required.

### Treatment

Treatment of hepatitis depends on the underlying etiology. Unfortunately, the etiology of the novel hepatitis remains unknown. Hence, supportive treatment is the foundation. Current research suggests that adenovirus infection appears to be an important factor, but there is no specific and proven effective treatment for adenovirus hepatitis. In terms of anti-viral therapy, cidofovir (CDV) may be a preferred agent. It is a cytosine nucleotide analog that inhibits DNA polymerase, and it works well *in vitro* but efficiency varies in clinical use (Cheng et al., 2020; Porritt et al., 2021; Brodin, 2022). In the report of Alabama, two of the cases that developed acute liver failure were treated with CDV and the clinical effect is unsatisfactory (Baker et al., 2022). The efficiency of CDV is unclear. In addition, corticosteroid therapy has been studied in indeterminate PALF. Some cases improved. But the survival may be relatively low (Molleston et al., 2008; Devictor et al., 2011; Chapin et al., 2019). Glucocorticoids produce broad anti-inflammatory effects and suppress the excess immune activation, which is considered to be the key disorder in acute severe hepatitis of unknown etiology. Therefore, glucocorticoids may be useful in certain patients, particularly in those who deteriorate rapidly. However, the risks and benefits of corticosteroid therapy should be balanced. Moreover, studies in pediatrics have shown plasmapheresis, and at times, in combination with other therapies, may serve as a bridge to liver transplantation (Squires et al., 2022). Finally, liver transplantation should be considered for patients with a poor prognosis. Nevertheless, different countries and regions have discrepant capacities for liver transplantation. Thus, identifying the etiology early and specific treatment for the pathogen is crucial, which may improve the survival rate and reduce the need for liver transplantation.

### Prevention

The infection of adenovirus or other pathogens cannot be excluded nowadays. Considering that adenovirus transmits through fecal-oral and respiratory routes, good hygienic practices (including careful hand hygiene, cleaning, and disinfection of surfaces) and social distancing among children will contribute to reducing the spread of the disease. Attention to food and water hygiene is also very effective and necessary.

## Conclusion

The etiology of acute hepatitis in children remains unknown. Infection markers of viruses have not been detected in any of the cases over the world. Adenovirus was detected in most cases and was regarded as one of the candidates’ underlying causes. Several hypotheses, including multisystem inflammatory syndrome and superantigen activation, have been proposed. An immune deficiency in children resulting from lack of exposure to pathogens because of the social distancing has rendered them more susceptible to normal adenovirus infection. An exceptionally large wave of normal adenovirus infections or a novel variant of adenovirus will cause rarer and more severe outcomes. Other infectious causes, including SARS-CoV-2, are being investigated. Acute hepatitis in children may be the post-infectious sequelae of SARS-CoV-2. Metagenomics sequencing analysis from cases in England and Scotland showed that other pathogens, such as AAV2, HHV6, HHV7, and human polyomavirus, have been detected. However, the relevant significance is under investigation. There are some limitations in the prior case reports all over the world. Firstly, the different definitions used at the beginning led to some bias in the diagnosis of patients. Secondly, due to uneven medical conditions in different countries, some tests cannot be launched in some countries, and the determination of the etiology is challenging. Although there is no case reported in several countries, precautions should be prepared ahead of time. Clinicians should be trained to raise awareness of this severe hepatitis of unknown etiology among young children. The reports of these cases prompt to perform syndrome-based surveillance at a local or a country scale and can obtain a more precise and continuous picture of the epidemiological and clinical features of infectious diseases. So far, there is no specific and effective treatment as the underlying cause is unclear. The priority is to identify the etiology of the novel epidemic and to further refine control and prevention measures of the syndrome.

## Author contributions

MZ: drafting of the manuscript. LC: design and critical revision of the manuscript. Both authors contributed to the article and approved the submitted version.
